# Primary pigmented nodular adrenocortical disease presenting with a unilateral adrenocortical nodule treated with bilateral laparoscopic adrenalectomy: a case report

**DOI:** 10.1186/1752-1947-4-230

**Published:** 2010-07-29

**Authors:** George N Zografos, Theodora Pappa, Spiros Avlonitis, Athina Markou, Dimosthenis T Chrysikos, Gregory Kaltsas, Chrysanthi Aggeli, George Piaditis

**Affiliations:** 1 Third Department of Surgery, Athens General Hospital "G. Gennimatas", Athens, Greece; 2 Department of Endocrinology and Diabetes Center, Athens General Hospital "G. Gennimatas", Athens, Greece

## Abstract

**Introduction:**

Primary pigmented nodular adrenocortical disease is a rare cause of adrenocorticotropic hormone-independent Cushing's syndrome. We report an uncommon primary pigmented nodular adrenocortical disease case presenting with a unilateral adrenocortical nodule and provide a brief overview of the existing literature.

**Case presentation:**

A 27-year-old Caucasian woman was admitted to our Department with adrenocorticotropic hormone-independent Cushing's syndrome. Its cause was initially considered a left adrenocortical adenoma based on computer tomography imaging. The patient underwent left laparoscopic adrenalectomy and histological examination revealed pigmented micronodular adrenal hyperplasia. Evaluation for the presence of Carney complex was negative. Six months later recurrence of hypercortisolism was documented and a right laparoscopic adrenalectomy was performed further establishing the diagnosis of primary pigmented nodular adrenocortical disease. After a nine-year follow-up there is no evidence of residual disease.

**Conclusions:**

Even though primary pigmented nodular adrenocortical disease is a rare cause of Cushing's syndrome, it should be included in the differential diagnosis of adrenocorticotropic hormone-independent Cushing's syndrome, especially because adrenal imaging can be misleading mimicking other adrenocortical diseases. Bilateral laparoscopic adrenalectomy is the preferred treatment in these subjects.

## Introduction

Primary pigmented nodular adrenocortical disease (PPNAD) and adrenocorticotropic hormone (ACTH)-independent macronodular adrenal hyperplasia (AIMAH) account for approximately 10% of ACTH-independent Cushing's syndrome (CS) [1-3].

PPNAD is characterized by pigmented adrenocortical nodules ranging in size from sub-microscopic to 10 mm in diameter. The cortical nodules are unencapsulated and appear black and brown containing large, globular cells with pigment-laden, eosinophilic cytoplasm, whereas the inter-nodular cortex is usually atrophic [4-6].

Half of PPNAD patients appear to be sporadic cases and the other half are familial, mostly associated with Carney complex (CNC) [4,7,8].

PPNAD may manifest with typical signs of CS or present with sub-clinical or cyclic CS [4,8]. The treatment of choice in CS due to PPNAD is bilateral adrenalectomy.

## Case presentation

A 27-year-old Caucasian woman was referred to our Surgery Department from the Endocrinology Department for surgical treatment of ACTH-independent CS considered to be caused by an adrenocortical adenoma.

The patient reported a three-year history of hirsutism, acne, menstrual disturbances and mood disorders. On clinical examination, skin atrophy, buffalo hump, moon facies, proximal muscle weakness and elevated blood pressure (145/90 mmHg) were documented. Our patient's history and clinical signs were suggestive of hypercortisolism and she was further evaluated.

Hormonal investigation revealed elevated morning cortisol levels (702, reference range (RR): 130-690 nmol/L) with suppressed ACTH levels (3.6, RR: 9-52 pg/mL), elevated urinary free cortisol (UFC) levels (221, RR: 20-90 μg/24 h), loss of circadian rhythm of cortisol secretion (plasma cortisol (16:00): 693 nmol/L, (00:00): 694: 694 nmol/L) and failure to suppress endogenous plasma cortisol following low dose dexamethasone suppression test (LDDST) (plasma cortisol after LDDST: 800 nmol/L) (Table 1).

**Table 1:** Hormonal investigation.

Plasma hormones	Results	Reference range
Cortisol	08:00: 702	Morning:130-690,
	16:00: 693	Evening: 70-345 nmol/L
	00:00: 694	

ACTH	3.2	9-52 pg/ml

UFC	221	20-90 μg/24 h

Cortisol following LDDST	800	<50 nmol/L

A 2 cm adenoma of the left adrenal gland was identified in the adrenal computerized tomography (CT) scan, whereas the right adrenal appeared normal in size and architecture.

**Figure 1:**
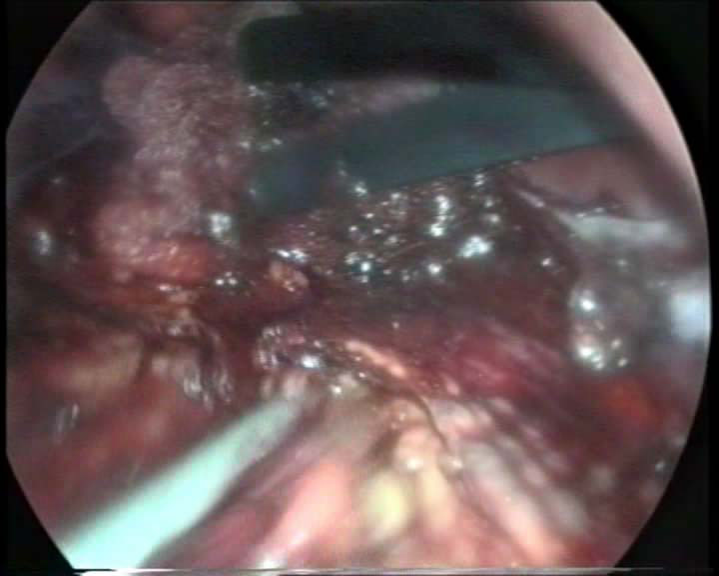
**Macroscopic pigmented micronodular appearance of adrenal glands on surgery**. Intra-operative view of the hyperplastic nodular adrenal glands.

The hormonal and radiological findings led to the diagnosis of ACTH-independent CS due to an adrenocortical adenoma of the left adrenal gland and our patient underwent left laparoscopic adrenalectomy. On surgery, both adrenals had macroscopically a pigmented, micronodular appearance (Figure 1). Histology revealed adrenal hyperplasia with small, pigmented cortical nodules establishing the diagnosis of PPNAD.

In order to exclude the presence of CNC, our patient underwent a chest CT scan, pituitary magnetic resonance imaging (MRI) scan, ultrasound (US) of the genitals and heart, and all were unremarkable. Her family history was negative.

Six months later recurrence of hypercortisolism was documented and a right laparoscopic adrenalectomy was performed. Histology confirmed the initial diagnosis of pigmented micronodular adrenal hyperplasia.

Our patient's post-operative course was uncomplicated and she received replacement therapy with hydrocortisone and fludrocortisone. On one-year follow-up, all signs and symptoms of CS resolved and nine years later there is no evidence of hypercortisolism or CNC.

### Technical aspects of laparoscopic adrenalectomy

We prefer the trans-peritoneal lateral decubitus approach as the best for maximal exposure of the gland and adjacent organs and vessels. In the right, we use three 10 mm trocars, introducing a fourth 5 mm at a later stage. In the left, we use two 10 mm trocars and two 5 mm trocars.

#### Right adrenalectomy

The right triangular ligament and the retroperitoneal liver attachments are cauterized and divided in order to allow liver retraction and expose the upper limits of the adrenal gland. After dividing the retroperitoneum, the inferior vena cava (IVC) is identified and dissected from the gland. The periadrenal fat is gently pushed upwards with endo-peanuts. The adrenal vein is subsequently identified, dissected, double-clipped and divided. The inferior and superior adrenal vessels are cauterized or clipped. Ultrasonic scissors are used after the ligation of the adrenal vein.

#### Left adrenalectomy

The left colonic flexure is occasionally mobilized and the left upper renal pole is exposed. The splenic attachments are cauterized and divided, and the tail of pancreas is identified. The spleen is further mobilized until the stomach is visualized. Gerota's fascia is then opened, the adrenal gland identified and the adrenal vein dissected, double clipped and divided. The upper adrenal vessels are either cauterized or clipped.

The specimen is placed in a bag and extracted after minimally extending the 10 mm port-site incision.

## Discussion

We describe a PPNAD case presenting with a large cortical nodule, mimicking a unilateral adrenocortical adenoma, thus illustrating the puzzling differential diagnosis of ACTH-independent CS.

In PPNAD the adrenal glands are usually normal in size, in some patients micronodules are visible and, even rarer, one or more macronodules (>1 cm in diameter) can be present uni- or bilaterally, making the differential diagnosis from AIMAH very difficult [9]. Bilateral uptake of iodocholesterol is demonstrated in cortical adrenal scintigraphy in most subjects with PPNAD [6]. The imaging of a solitary cortical nodule on CT scan with otherwise normal adrenal glands made the diagnosis of bilateral adrenocortical dysfunction remote and no iodocholesterol scintigraphy was performed.

Even though our patient presented with typical signs and symptoms of CS (central obesity, hirsutism, myopathy, hypertension), the clinical picture of PPNAD may also be subtle or cause cyclical CS, i.e. episodes of cortisol excess interspersed by periods of normal cortisol secretion [4,8]. These atypical manifestations of the disease may lead to further delay in diagnosis and treatment.

Patients with PPNAD fail to suppress cortisol by LDDST and high dose dexamethasone suppression test (HDDST) and the majority of them characteristically respond with an increase of UFC by 100% or more using the Liddle's test (sequential LDDST and HDDST). This test has been useful to differentiate PPNAD from AIMAH or identify asymptomatic subjects with CNC [10]. This delayed paradoxical response was associated with an increased expression of the glucocorticoid receptor (GR); its molecular basis still remains to be clarified [11].

Once diagnosis of PPNAD was established histologically, our patient underwent evaluation for CNC, since half of PPNAD cases are sporadic and the other half familial, usually associated with CNC [4,7]. The latter is a multiple endocrine neoplasia consisting of spotty skin pigmentation, cardiac myxomas and endocrine over-activity (mainly hypercortisolism and/or growth hormone overproduction) [4,7,8]. PPNAD is observed in 25% of CNC subjects [4,7]. Almost 50% of CNC patients are familial cases. One of the putative genetic loci, mapped on chromosome 17q22-24, has been identified as the type 1α regulatory subunit of cyclic adenosine monophosphate (cAMP)-dependent protein kinase A (PRKAR1α) [12-15]. PRKAR1α inactivating mutations were found in approximately half of CNC kindreds [15]. Linkage analysis has also identified a putative genetic locus at chromosome 2p16 [12,13]. Genetic testing was not performed in our patient.

Recurrence of hypercortisolism six months post-operatively led to completion of bilateral adrenalectomy further supporting the diagnosis of PPNAD. Even though there is a report of a PPNAD subject undergoing unilateral adrenalectomy without recurrence of clinical CS, the long-term follow-up of this patient demonstrated abnormal cortisol secretion [16]; on the other hand, there are follow-up studies, where subtotal or unilateral adrenalectomy in some PPNAD cases resulted in remission of hypercortisolism [6,17].

Bilateral adrenalectomy is the treatment of choice for CS due to PPNAD. The laparoscopic approach is associated with lower morbidity rate compared with the open technique, less post-operative pain, shorter hospitalization time and lower overall cost.

On nine-year follow-up our patient has no clinical or biochemical signs of CS, whereas periodic evaluation for CNC remains negative.

## Conclusions

Even though PPNAD, sporadic or familial, is a rare cause of CS, it should be included in the differential diagnosis of ACTH-independent CS, especially because adrenal imaging can be misleading mimicking other adrenocortical diseases. Every patient diagnosed with PPNAD should be screened for CNC and monitored closely on a long-term basis. Bilateral laparoscopic adrenalectomy is the preferred treatment in subjects with PPNAD.

## Consent

Written informed consent was obtained from the patient for publication of this case report and any accompanying images. A copy of the written consent is available for review by the Editor-in-Chief of this journal.

## Competing interests

The authors declare that they have no competing interests.

## Authors' contributions

GZ, SA, DC and CA analyzed and interpreted the patient's surgical data. TP was a major contributor in writing the manuscript. AM contributed substantially to the revised editing of the report. GK and GP provided critical assistance for the intellectual content of this manuscript. All authors read and approved the final manuscript.
